# Identification of a novel *ST3GAL5* variant in a Chinese boy with GM3 synthase deficiency and literature review of variants in the *ST3GAL5* gene

**DOI:** 10.1186/s13023-024-03369-6

**Published:** 2024-11-12

**Authors:** Dan Mu, Yanting Yang, Yao Liu, Ying Shen, Hongqian Liu, Jing Wang

**Affiliations:** 1 Department of Obstetrics and Gynecology, West China Second University Hospital, Sichuan University, Chengdu, 610041 China; 2 Department of Medical Genetics / Prenatal Diagnostic Center, West China Second University Hospital, Sichuan University, Chengdu, 610041 China; 3 Key Laboratory of Birth Defects and Related Diseases of Women and Children (Sichuan University), Ministry of Education, Chengdu, 610041 China; 4 Department of Obstetrics/Gynecology, Joint Laboratory of Reproductive Medicine (SCU-CUHK), West China Second University Hospital, Sichuan University, Chengdu, 610041 China

**Keywords:** Compound heterozygous variations, GM3 synthase deficiency, Gangliosides, *ST3GAL5* gene, Trio-whole exome sequencing

## Abstract

**Background:**

GM3 synthase deficiency (GM3SD) is an autosomal recessive disorder resulting from mutations in the *ST3GAL5* gene. It is characterized by intellectual disability, microcephaly, psychomotor and developmental delay, hearing and visual impairments, and changes in skin pigmentation. This study aims to broaden the genetic mutation spectrum of GM3SD through the report of a de novo mutation and a comprehensive summary of GM3SD phenotype to aid in genetic counseling and prenatal diagnosis.

**Results:**

Compound heterozygous variants in *ST3GAL5* (NM_003896.4: c.1000delC, p.Arg334Glufs*15 and c.207-1G > T, p.Cys70Glufs*81) were identified via trio-whole exome sequencing (trio-WES) and confirmed pathogenic through functional experiments. Notably, c.207-1G > T was a newly discovered variant. Additionally, previously reported GM3SD mutations were classified into R288X and non-R288X, revealing that R288X mutations were more likely to manifest developmental, emotional abnormalities, and severe feeding difficulties.

**Conclusions:**

This study reveals a novel mutation in *ST3GAL5* and provides a comprehensive overview of GM3SD phenotype, aiding in the diagnosis and genetic counseling of GM3SD in clinical practice.

## Introduction

GM3 synthase deficiency (GM3SD; OMIM 609056), also known as salt and pepper development regression syndrome (SPDRS), is an autosomal recessive disorder caused by homozygous or compound heterozygous mutations in the ST3 Beta-Galactoside Alpha-2,3-Sialyltransferase 5 (*ST3GAL5*) gene located at 2p11.2 [1]. The identification of the *ST3GAL5* gene as a pathogenic locus in GM3SD originated from research within the Amish population in 2004. The *ST3GAL5* gene encodes lactosylceramide alpha-2,3-sialyltransferase (GM3 synthase), responsible for catalyzing the transfer of sialic acid to lactosylceramide, thus synthesizing the ganglioside GM3 [2]. Ganglioside GM3 participates in signal transduction, cell proliferation regulation, induction of cell differentiation, maintenance of fibroblast morphology, and integrin-mediated cell adhesion [3]. Gangliosides, prevalent in the central nervous system, play pivotal roles in brain development and function. Among the dominant gangliosides in the mammalian brain are GD1a, GD1b, GM1, and GT1b. GM3 serves as a precursor to several brain gangliosides, including GD1a, GD1b, GM1, and GT1b [4]. Mutations in the *ST3GAL5* gene result in GM3 synthase dysfunction, leading to the deficiency of GM3 and its downstream substances. Consequently, GM3SD may precipitate severe neurological disorders.

Detecting GM3SD during pregnancy presents challenges. Newborns with GMSD typically exhibit normal birth parameters but progressively develop disease-related manifestations over time. Early signs include irritability, feeding difficulties, necessitating gastrostomy tube (G-tube) placement for growth, intractable seizures, acquired microcephaly, hypotonia, and sensory impairments [1, 2, 5]. As they grow, affected children often experience developmental delays, severe intellectual disabilities, and may display skin pigmentation changes on their extremities [1, 5]. Studies indicate that children with GM3SD are prone to otitis media, pneumonia, and progressive scoliosis [6]. Median age at death, primarily due to pneumonia, is reported as 23.5 years [6]. Treatment for GM3SD is symptomatic, supportive, and tailored to individual needs. While some clinical features persist, others vary in expression. Antiepileptic drugs are prescribed for seizures, and long-term feeding difficulties may necessitate G-tube placement. Hearing loss can be managed with hearing aids. Although oral ganglioside supplementation temporarily alleviates symptoms, it does not alter disease progression [7]. Regular follow-up is crucial for monitoring growth parameters, developmental progress, and detecting new clinical manifestations. Patients should undergo routine assessments for vision, hearing, scoliosis, and pneumonia. Currently, clinical diagnostic criteria for GM3 synthetase deficiency are lacking, necessitating reliance on molecular genetic testing for diagnosis.

GM3SD prevalence is notably high among the Amish population, with an estimated incidence of homozygous c.862C > T (p.Arg288Ter) variant in the *ST3GAL5* gene at approximately 1 per 1200 births among the Amish [8]. While additional variants have been identified in non-Amish populations, the carrier frequency in the Amish population is estimated at 5–6% [6, 9]. In this study, we present a case of GM3SD diagnosed in a 4-years-old child at 22 months of age. Trio-whole exome sequencing (trio-WES) revealed compound heterozygous variants in ST3GAL5 (NM_003896.4: c.1000delC, p.Arg334Glufs*15 and c.207-1G > T, p.Cys70Glufs*81) with confirmation of pathogenicity, including the identification of c.207-1G > T as a novel variant. Our identification of this novel variant expands the mutational spectrum of the ST3GA*L5* gene. We conducted a comprehensive review of mutational spectrum, and genotype–phenotype correlations in GM3SD to enhance genetic counseling and diagnosis. Given the rarity of GM3SD, existing literature predominantly comprises case reports, reports of novel mutations, and associated clinical manifestations, primarily among the Amish. While the R288X mutation is prevalent in the Amish, new mutations have been identified in other ethnic groups. To date, there is limited exploration into the relationships between different mutations and phenotypes. In our study, we categorized reported mutations into R288X and non-R288X to compare common phenotypes and lay the groundwork for future investigations into the relationship between mutations and phenotypes.

## Materials and methods

The patient and his parents were enrolled at West China Second University Hospital of Sichuan University. This study received approval from the Ethical Review Board of West China Second University Hospital (IRB no. 2019(040)), Sichuan University. Written informed consent was obtained from each participant or their guardian.

### Genetic studies

Genomic DNA was isolated from peripheral blood samples of the proband and his parents using the FitAmp Plasma/Serum DNA Isolation Kit (Epigentek Exon). Exon capture and DNA sequencing were performed using the Agilent SureSelect Human All Exon V6 Kit for exon capture and the Illumina HiSeq X system (Illumina) for sequencing, following the manufacturer’s instructions. The reads were aligned to the reference genome (GRCh38) using the Burrows-Wheeler Aligner (BWA) software. Functional annotations were performed using the ANNOVAR software, and data filtering was conducted using HGMD, Clinvar, dbSNP, 1000 Genomes Project, gnomAD, and ExAC. Variant classification (pathogenic, likely pathogenic, uncertain, likely benign, and benign) followed the variant interpretation guidelines of the American College of Medical Genetics and Genomics (ACMG) [10–12]. Functional assessment of the variant and its correlation with the disease phenotype were conducted using data from the Online Mendelian Inheritance in Man (OMIM) database along with previously published literature [13, 14].

**Fig. 1:**
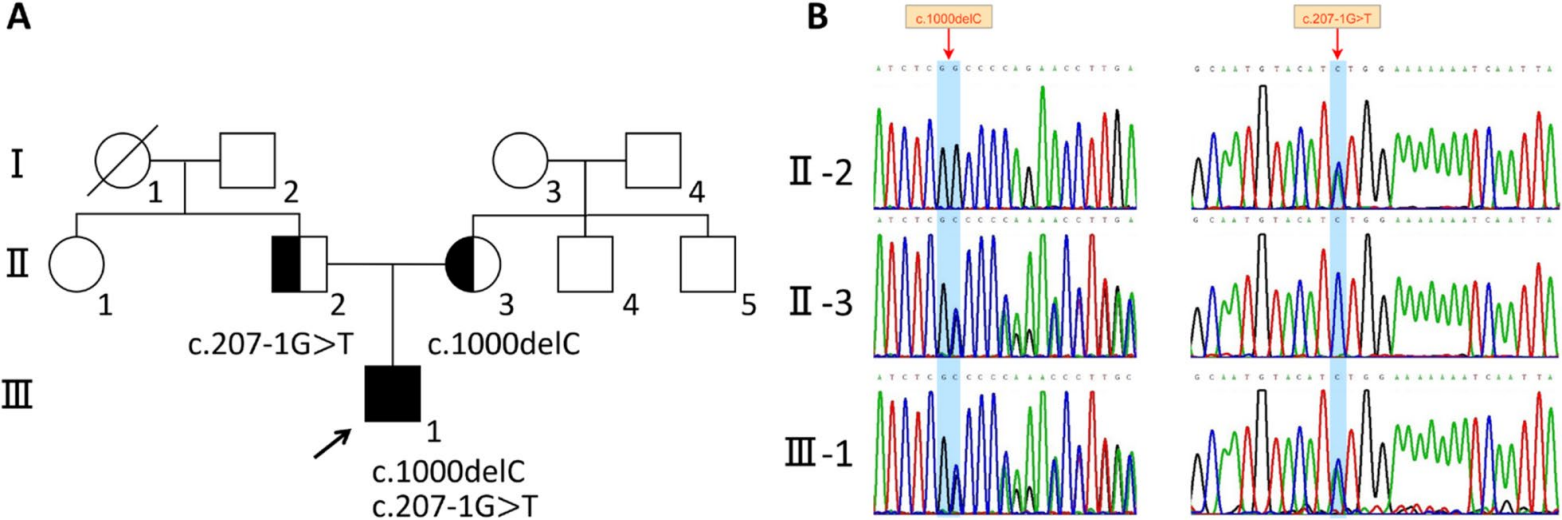
*ST3GAL5* compound heterozygous variant in the GM3SD family. **A** Family Pedigree: Pedigree of the family members. The arrow indicates the patient. Squares represent males, and circles represent females. **B** Sequence analysis of the human *ST3GAL5* gene: Sanger sequencing results showing that member III-1 had a compound heterozygote while the parents II-2 and II-3 were heterozygous carriers

Through trio-WES, two candidate pathogenic variants were identified in *ST3GAL5*, which were compound heterozygous variants (NM_003896.4: c.1000delC and c.207-1G > T), and then validated using Sanger sequencing. Polymerase chain reaction (PCR) amplification reactions were performed using the ProFlex PCR System (Thermo Fisher), and the products were sequenced on an ABI377A DNA Sequencer (Applied Biosystems). The primers used for Sanger sequencing are F: 5′-GTGTTAATGTGCTGCCTAC-3′ and R: 5′-GCCTTGGTCTGATGAGTG-3′ (c.1000delC); F: 5′-CATGTCACATTCTTCAGTAG-3′ and R: 5′- CATAGCAGGCAGACTCATT-3′ (c.207-1G > T) (Fig. 1).

### Cell culture and transient transfection

HEK293T cells were obtained from the American Type Culture Collection (ATCC) and cultured in DMEM supplemented with 10% fetal bovine serum and 1% penicillin–streptomycin. Cultured cells were maintained in a sterile incubator with 5% CO_2_ at 37 °C. Cells at the third passage were equally distributed into six-well plates, and plasmid transfection was performed when cell confluency reached 70–80%. Plasmids carrying the wild type and variant c.1000delC were transfected using jetPRIME® reagent according to the manufacturer’s instructions.

### RNA extraction and quantitative real-time PCR

**Fig. 2:**
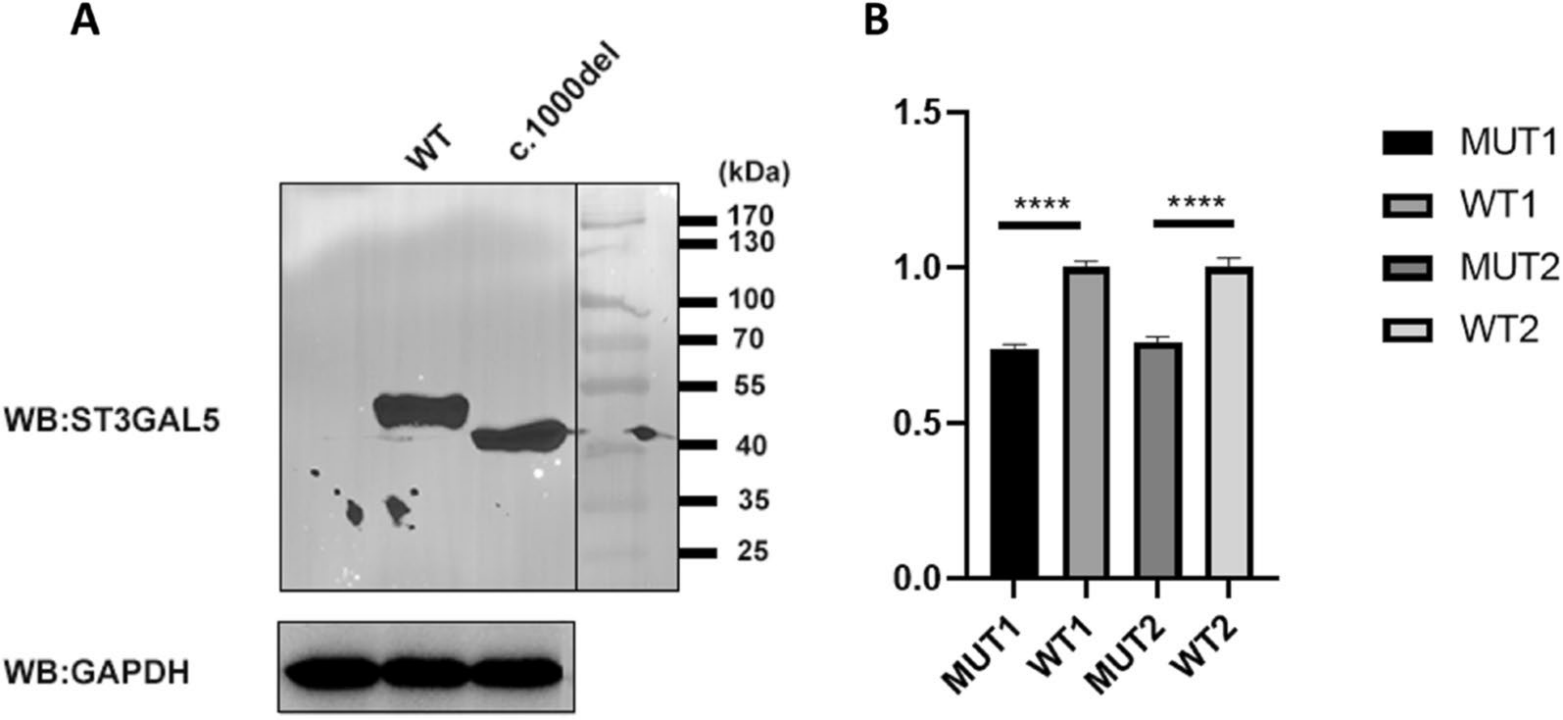
Effect of the variant c.1000delC on ST3GAL5 protein and mRNA levels in HEK293T cells. **A** Western blotting results: The wild-type sample showed a ~ 50-KD band, while the protein produced by the R334E fs*15 variant is truncated, showing a ~ 47-KD band. **B** qPCR results of the c.1000delC variant: Two qPCR experiments were performed. The first result (MUT1, WT1) decreased by about 26%, and the second result (MUT2, WT2) decreased by about 24%. The primer sequences for the two qPCR experiments were as follows: F1: 5′-GCATTATGTGGACCCTGAC-3′ and R1: 5′-TTGGCAAACTTGGGACGA-3´; F2: 5′-AACCCAGAACACCTTTGCAC-3′ and R2: 5′-TCACCACTCCCTCTTTGACC-3′

RNA extraction was performed from HEK293T cells 24 h after plasmid transfection using TRIzol reagent. The cells were centrifuged at 10,000×*g* for 10 min and precipitated using isopropanol. The precipitated RNA was collected by centrifugation at 10,000×*g* for 10 min at 4 °C. Precipitated RNA was extracted using phenol. Finally, RNA was precipitated using 75% ethanol. Reverse transcription of extracted RNA to cDNA was carried out using Hiscript III Reverse Transcriptase (Vazyme), followed by qPCR using Green Premix Ex Taq II. GAPDH expression served as the reference, and the results were analyzed using the E^−△△CT^ method (Fig. 2).

### Western blotting analysis

Proteins were extracted from cultured HEK293T cells 48 h after plasmid transfection using a universal protein extraction lysis buffer containing a protease inhibitor cocktail. Protein concentration was determined using a BCA protein quantification kit. Denatured proteins were separated on 10% SDS–polyacrylamide gels and transferred to a polyvinylidene fluoride (PVDF) membrane. Membranes were probed with anti-Flag (1:1000, Abcam; mouse) and anti-GAPDH (1:5000, Abcam; rabbit) primary antibodies. And we use goat anti-mouse secondary antibody (1:50000) or goat anti-rabbit secondary antibody (1:50000) as the second antibodies (Fig. 2).

### Minigene construct, transfection and RT-PCR

**Fig. 3:**
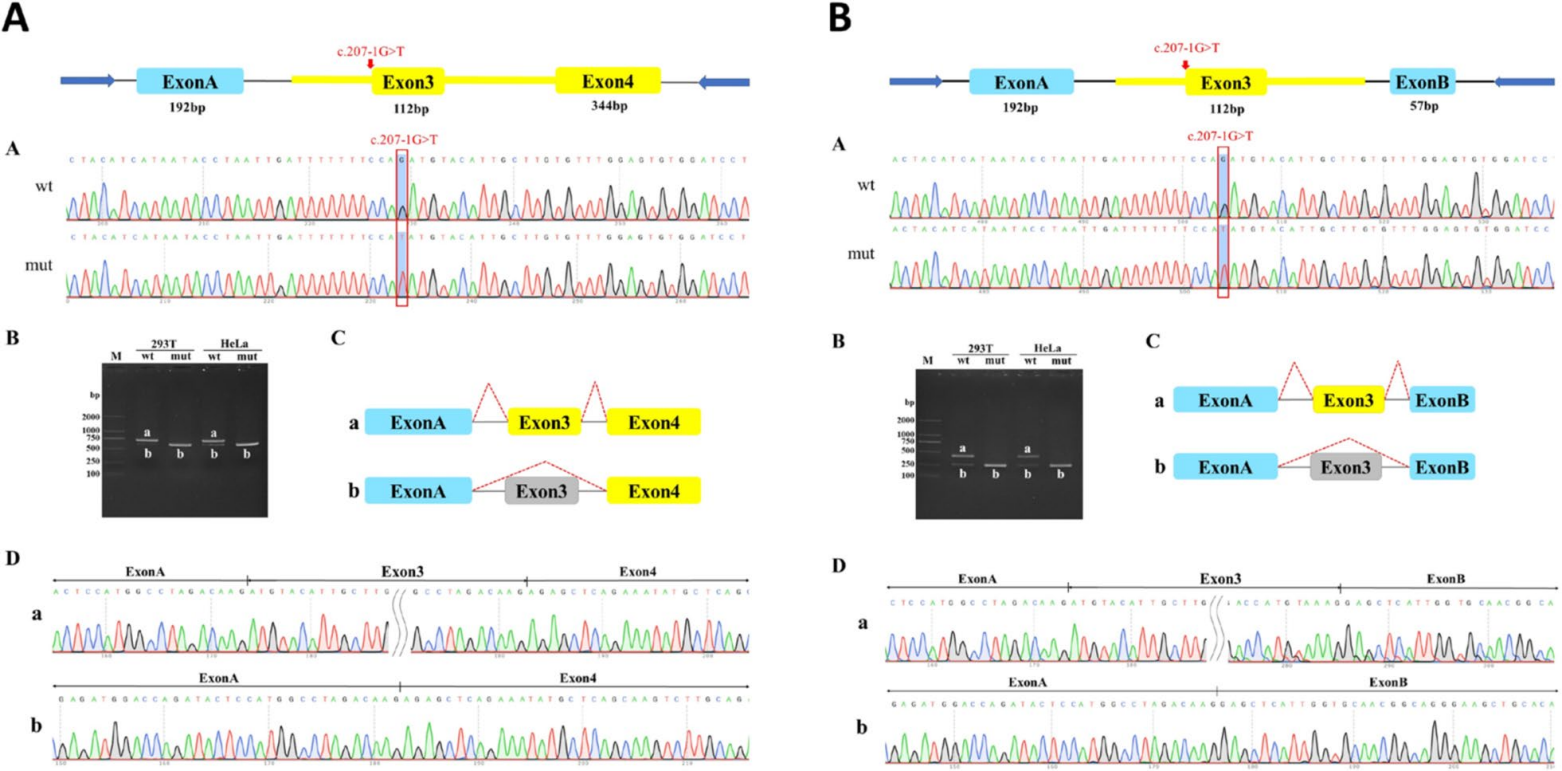
Functional effects of the variant c.207-1G > T on *ST3GAL5* gene transcript. **A** Results of pcMINI-C vector: In HeLa and 293 T cells, the wild-type had two bands, with the large band consistent with the expected size (716 bp), named band a, and the small band named b. The wild-type bands from the two cell lines were subjected to TA cloning followed by Sanger sequencing. The mutant type in HeLa and 293 T cells had a single band, named band b, and the mutant bands from the two cell lines were subjected to Sanger sequencing. The wild-type strip a is a normal shear strip with Exon A (192 bp)-Exon 3 (112 bp)-Exon 4 (344 bp); the wild-type strip b is an abnormally sheared strip with Exon 3 jumping and Exon A (192 bp)-Exon 4 (344 bp). The mutant strip b is an abnormally sheared strip with Exon 3 jumping, sheared as Exon A (192 bp)-Exon 4 (344 bp). **B** Results of pcMINI vector: In HeLa and 293 T cells, the wild-type had two bands, with the large band consistent with the expected size (384 bp), named band a, and the small band named b. The wild-type bands from the two cell lines were subjected to TA cloning followed by Sanger sequencing. The mutant type in HeLa and 293 T cells had a single band, named band b, and the mutant bands from the two cell lines were subjected to Sanger sequencing. Wild-type strip a is a normal shear strip with Exon A (192 bp)-Exon 3 (112 bp)-Exon B (57 bp); wild-type strip b is an abnormally sheared strip with Exon 3 jumping and Exon A (192 bp)-Exon B (57 bp). Mutant band b is an abnormally sheared band, Exon 3 jumping, sheared in Exon A (192 bp)-Exon B (57 bp)

Functional splicing reporter minigene assays were employed to evaluate the effects of sequence variants on splicing. Four vectors were constructed: pcMINI-wt, pcMINI-mut, pcMINI-C-wt, and pcMINI-C-mut. Sanger sequencing confirmed the successful insertion of both wild-type and mutant minigenes into their corresponding vectors, resulting in four recombinant vectors. These vectors were transfected into HeLa and 293 T cell lines and RNA extraction was performed on eight samples collected 48 h post-transfection. The pcMINI-wt/mut-*ST3GAL5* minigene was created by inserting a partial intron 2-Exon 3-partial intron 3 sequence into the pcMINI vector containing the universal Exon A-intron A-MCS-intron B-Exon B structure. The minigene constructs pcMINI-C-*ST3GAL5*-wt/mut contained a partially intron2-Exon3-partially intron3-Exon4 sequence inserted into a pcMINI-C vector containing the universal Exon A-intron A-MCS structure. Splicing patterns of the wild-type and mutant transcripts were compared using RT-PCR and sequencing following transient transfection into 293 T and HeLa cells (Fig. 3).

## Results

### Case presentation

The patient was born to nonconsanguineous Chinese parents without apparent abnormalities. Neonatal jaundice was observed at birth, and promptly treated with blue-light therapy. At 3 months old, the child experienced a high fever of 39 °C, followed by generalized rigidity, clenched fists, staring eyes, and cyanosis of the lips, lasting 1 min and resolving spontaneously. By 5 months old, the child displayed poor interactions, weighing 6 kg (< 3rd percentile), with a length of 62.7 cm (> 3rd percentile), and a head circumference of 37.5 cm (< 3rd percentile). The fontanel remained open, and muscle strength and tone were diminished. Laboratory tests indicated increased blood amino acid metabolism, hyperlactatemia, and poor urea cycle function. Urine organic acid analysis revealed non-ketotic dicarboxylic aciduria, hepatic damage, and insufficient energy production. Cranial MRI showed a slight widening of the extracerebral space in the bilateral frontotemporal region. Chest radiography and CT indicated pneumonia and gastroesophageal ultrasound suggested gastroesophageal reflux. Audio-visual evoked potentials were abnormal, with increased ABR response thresholds in both ears. EEG findings were abnormal. Cranial CT, liver ultrasound, gallbladder ultrasound, pancreas ultrasound, spleen ultrasound, kidney ultrasound, and electrocardiography showed no abnormalities. Treatment with oxcarbazepine had limited efficacy. The patient was diagnosed with developmental delay, microcephaly, epilepsy, hyperlactatemia, and non-ketotic dicarboxylic aciduria. At 6 months old, the child exhibited hair thinning, abdominal distension, and an umbilical hernia. At 22 months old, the child returned to the hospital with a height of 75 cm (< 3rd percentile), weight of 6 kg (< 3rd percentile), and head circumference of 41 cm (< 3rd percentile). The patient experienced sudden shouting during sleep, fatigue, poor visual and auditory tracking abilities, unsteady neck, inability to crawl, sit, stand, or walk without support, nonverbal communication, constipation, inability to grasp objects, express needs, or follow simple commands, and lagged peers in cognitive and comprehension skills. These symptoms were consistent with previously reported clinical manifestations of GM3SD.

At 6 months old, the patient started oral oxcarbazepine for epilepsy. The patient is now 4 years old, and the frequency of the patient’s epileptic seizures has decreased from a maximum of three times a day at the beginning to only one occurrence in the past 2 years. The patient continued to experience dystonia and constipation, but with good parental care, feeding difficulties improved significantly, and pneumonia frequency decreased.

### Identification of compound heterozygous mutations in the *ST3GAL5* gene

Trio WES analysis was conducted on the proband and his parents, revealing compound heterozygous variants in *ST3GAL5* (c.1000delC, p.Arg334Glufs*15 and c.207-1G > T, p.Cys70Glufs*81). Sanger sequencing of the patients and their parents confirmed that the mother carried a heterozygous variant of c.1000delC, while the father carried a heterozygous variant of c.207-1G > T (Fig. 1). Both heterozygous mutations were classified as likely pathogenic according to the ACMG guidelines.

### Impairment of *ST3GAL5* expression by compound heterozygous mutations c.1000delC and c.207-1G > T

To further elucidate the deleterious effects of *ST3GAL5* mutations on its expression, in vitro experiments were performed. Expression vectors containing wild-type *ST3GAL5* and mutated *ST3GAL5* were constructed and transiently transfected into HEK293T cells. mRNA levels of *ST3GAL5* were analyzed, revealing decreased expression in cells transfected with the mutated plasmid compared to the wild-type. Western blotting indicated that the protein produced by Arg334Glufs*15 was truncated, consisting of 348 amino acids, and confirmed to be pathogenic [2]. Conversely, the wild-type sample exhibited a ~ 50-KD band consistent with the expected molecular size of the ST3GAL5 protein, comprising 418 amino acid residues.

WES results suggested that the c.207-1G > T variant disrupts the original splice site. Minigene splicing experiments were conducted to validate the aberrantly spliced transcripts caused by this mutation. The results indicated that the c.207-1G > T mutation affected the normal splicing of *ST3GAL5*, resulting in an expression of c.207_318del, p.Cys70Glufs*81 due to exon 3 skipping. Overall deletion of exon 3 led to a change in the subsequent reading frame, producing a truncated protein.

## Literature Review

**Fig. 4:**
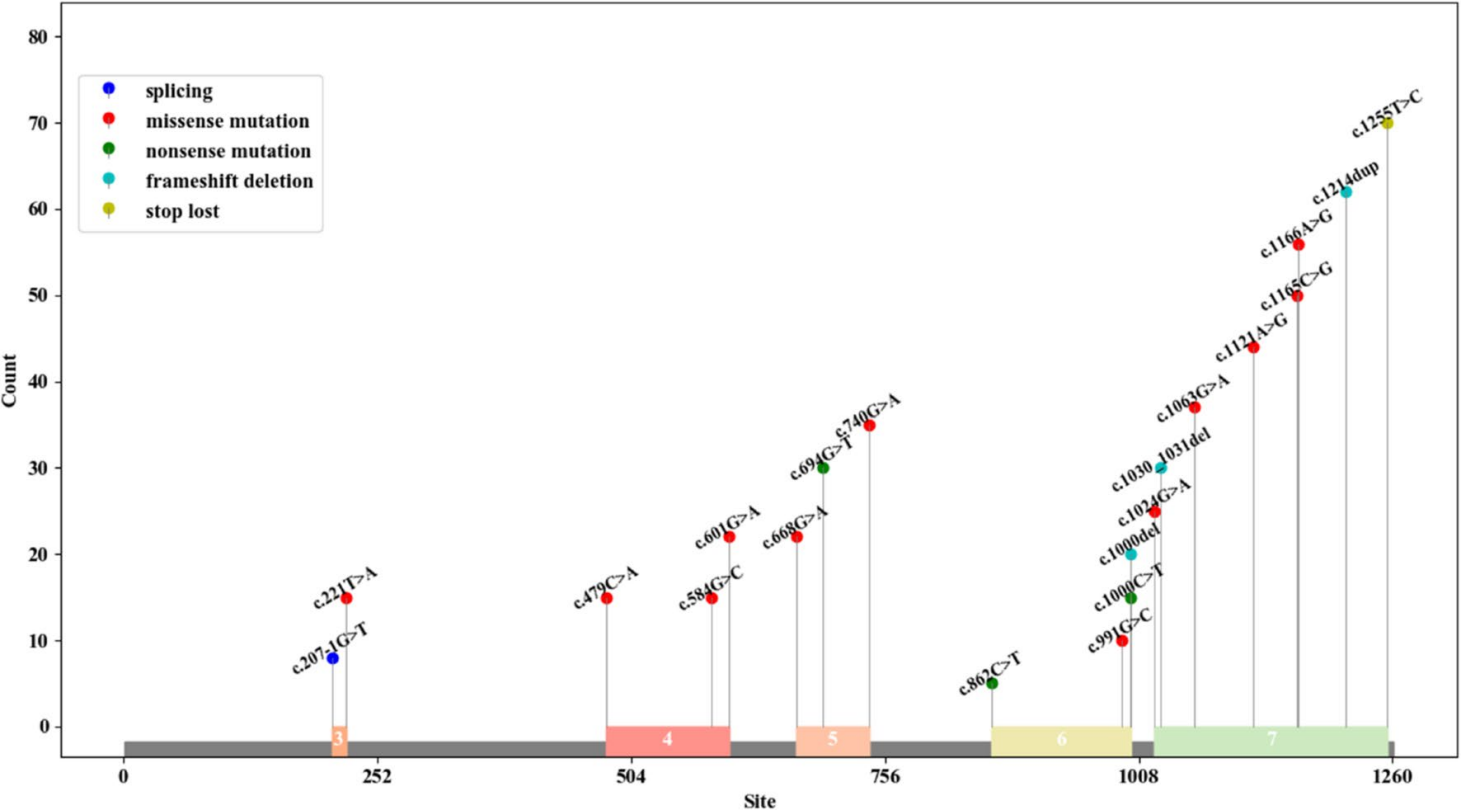
Previously reported mutations of the *ST3GAL5* gene and their exon locations

**Fig. 5:**
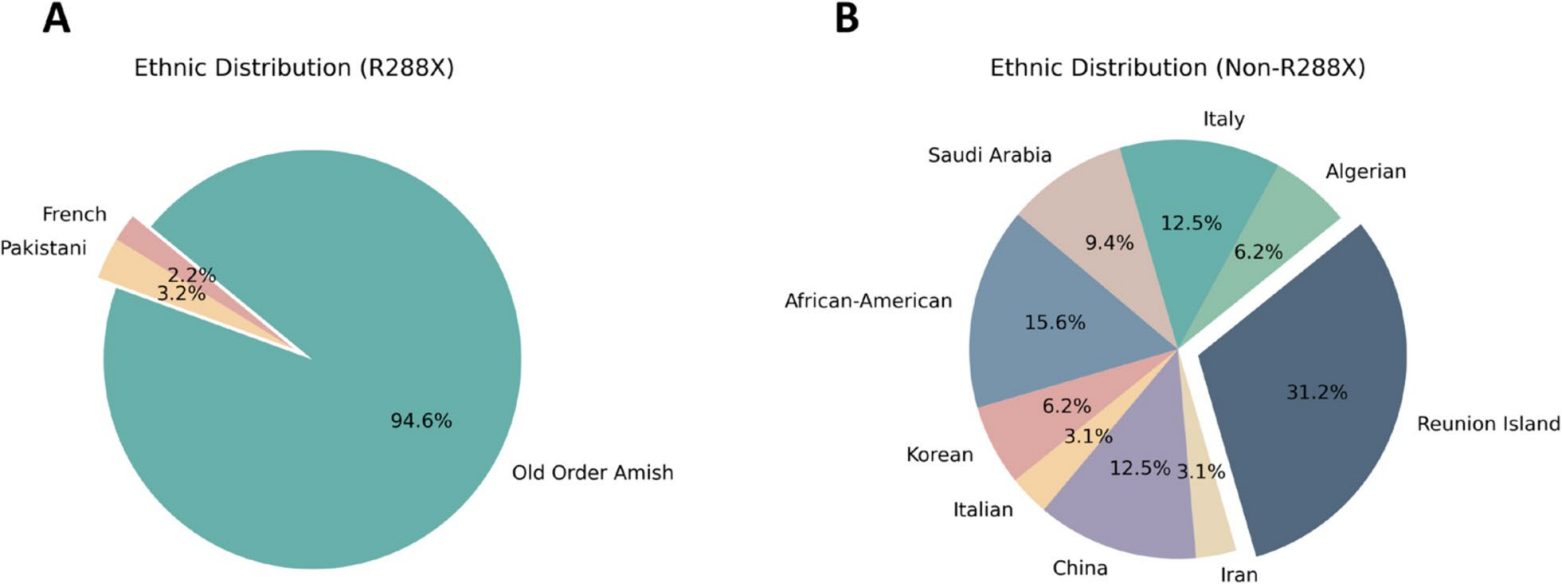
Ethnic distributions of R288X cohort and non-R288X cohort. **A** Ethnic distribution of patients in the R288X cohort. **B** Ethnic distribution of patients in the non-R288X cohort

We identified 15 studies comprising 125 patients. In the study by Wang et al. [15] 38 patients were reported, among whom 8 were also described by Simpson et al. [16]. Hence, Simpson et al. (2004) was excluded. We have compiled the common clinical signs of GM3 synthase deficiency (GM3SD), summarizing the clinical presentations reported in previous literature. The homozygous variant Arg288Ter (R288X) is frequently observed in the Amish population. However, Fragaki et al. [17] identified this variant in two French individuals, while Gordon-Lipkin et al. [18] found the same mutation in a Pakistani family, with three affected individuals. As per the reported literature, the R288X variant has been identified in 93 patients [6, 15, 17, 18]. Conversely, other mutations have shown dissemination, with various mutants detected across different ethnic groups. For instance, E355K was found in 4 African-American patients [19], C195S and G201R in 2 Korean patients [20], G342S in 1 Italian patient [21], D331N and E223G in 1 Chinese patient [22], I344Cfs*11 in 1 Iranian patient [23], Y374C and H389D in 1 African-American patient [24], G247D and E355K (compound heterozygote) and G247D (homozygous mutation) in 10 Reunion Island patients [3], X419RextX38 in 2 Algerian patients [3], R334X and H389R in 4 Italian patients [3], V74E in 3 Saudi Arabian patients [1], R334Efs*15 and V406Sfs*10 in 2 Chinese patients [2], and R334Efs*15 and C70Efs*81 in 1 Chinese patient, totaling 32 patients. We summarized the variants of *ST3GAL5* gene that have been reported previously, and the details were shown in Fig. 4. Based on the different mutations, we categorized patients into R288X and non-R288X cohorts for comparison of clinical manifestations, as depicted in Table 1 and Fig. 5.

**Table 1:** Comparison of phenotypes between R288X and non-R288X patients

Phenotypes	Group		Total
	R288X	Non-R288X	
Number of patients	93	32	125
Psychomotor delay	93/93(100%)	31/32(96.9%)	124/125(99.2%)
Microcephaly	53/93(57%)	18/32(56.3%)	71/125(56.8%)
Epilepsy	76/93(81.7%)	22/32(68.8%)	98/125(78.4%)
Dystonia/Movement disorder	55/93(59.1%)	25/32(78.1%)	80/125(64%)
Sit or walk independently	14/93(15.1%)	2/32(6.3%)	16/125(12.8%)
Developmental stagnation/failure to thrive	93/93(100%)^A^	7/32(21.9%)^B^	100/125(80%)
Development delay	91/93(97.8%)^C^	27/32(84.4%)^D^	118/125(94.4%)
Hearing impairment	28/93(30.1%)	13/32(40.6%)	41/125(32.8%)
Vision impairment	26/93(28.0%)	12/32(37.5%)	38/125(30.4%)
Abnormal pigmentation	29/93(31.2%)	14/32(43.8%)	43/125(34.4%)
Irritability	74/93(79.6%)^E^	16/32(50%)^F^	90/125(72%)
Feeding difficulties	36/93(38.7%)	16/32(50%)	52/125(41.6%)
Gastrostomy feeding tube	27/93(29.0%)^G^	3/32(9.4%)^H^	30/125(24%)
Facial dysmorphic features	0/93(0%)^I^	7/32(21.9%)^J^	7/125(5.6%)
Scoliosis	14/93(15.1%)	5/32(15.6%)	19/125(15.2%)
Abnormal electroencephalographic	43/93(46.2%)	9/32(28.1%)	52/125(41.6%)

A versus B: *P* = *p* = 0.000 (Pearson’s Chi-squared test); C versus D: *P* = 0.016 (Yates’ continuity correction of the Chi-squared test); E versus F: *P* = 0.001 (Pearson’s Chi-squared test); GvsH: *P* = 0.025 (Pearson’s Chi-squared test); I versus J: *P* = 0.000 (Yates’ continuity correction of the Chi-squared test)

We observed that both R288X and non-R288X patients exhibited psychomotor delay, microcephaly, epilepsy, dystonia/movement disorder, sit or walk independently, developmental stagnation/failure to thrive, development delay, hearing impairment, vision impairment, abnormal pigmentation, irritability, feeding difficulties, gastrostomy feeding tube, scoliosis, and abnormal electroencephalographic These may stem from GM3 synthase deficiency, leading to a deficit in several gangliosides, including GM3, common in both R288X and non-R288X patients. Developmental stagnation/failure to thrive, development delay, irritability, and gastrostomy feeding tube were more prevalent in R288X patients compared to non-R288X patients, whereas facial dysmorphic features more common in non-R288X patients. There was no significant statistical difference in the incidence of other phenotypes between the two groups of patients (*P* > 0.05). Detailed information is available in Table 1. Thus, we hypothesize that functional abnormalities induced by the R288X mutation are more likely to result in developmental, emotional abnormalities, and severe feeding difficulties. Additionally, differences in the occurrence of different mutations in various phenotypes warrant further investigation.

## Discussion

Gangliosides are ubiquitous on the cell surface of nearly all mammals, with particularly high concentrations in the nervous system. GM3, a ganglioside, along with its four derivatives—GM1, GD1a, GD1b, and GT1b—comprise over 90% of brain gangliosides. These molecules play pivotal roles in cell proliferation, adhesion, and various cellular processes. Mutations in the *ST3GAL5* gene leading to GM3 synthase deficiency result in severe phenotypes, including intellectual disability, developmental stagnation, microcephaly, hearing impairment, and visual impairment. While the precise mechanism underlying GM3 synthase deficiency remains elusive, researchers have observed significant reductions in GM3 and its downstream derivatives, alongside abnormal accumulation of lactosylceramide and its alternative metabolites in the plasma and fibroblasts of affected patients.

Since the initial report of GM3 synthase deficiency, also known as infantile-onset symptomatic epilepsy syndrome, in the Amish population in 2004, identical or distinct variants have been identified globally. Predominantly, affected individuals are from the Amish community, with the R288X variant being the sole identified variant in this population. Conversely, diverse variants have been identified in other populations, each dispersed. In this review, we consolidate previously reported variants of the *ST3GAL5* gene and their ethnic distributions, while analyzing the correlation between these variants and phenotypes. Based on our literature review, Developmental stagnation/failure to thrive, development delay, irritability, and gastrostomy feeding tube were more prevalent in R288X patients compared to non-R288X patients, whereas facial dysmorphic features more common in non-R288X patients. There was no significant statistical difference in the incidence of other phenotypes between the two groups of patients (*P* > 0.05). Variations in manifestations exist between R288X and non-R288X patients, suggesting potentially more severe phenotypes in R288X patients. Consequently, heightened attention should be directed toward the overall development, feeding status, and psychological condition in R288X patients. This review not only expands the mutation database of the *ST3GAL5* gene but also underscores the diversity of mutations in non-Amish populations, laying the groundwork for future mechanistic studies contributing to GM3 synthase deficiency.

The results of our qPCR experiments for the c.1000delC mutation were significant in both groups. Western blotting confirmed that the mutant protein was truncated. Therefore, we conducted an analysis of RNA degradation, selective mRNA transcription, and protein folding and modification, which may have contributed to an imbalance between the RNA and protein quantities produced. The minigene analysis results from our study suggest that the c.207-1G > T mutation impacts normal mRNA splicing, resulting in Exon3 skipping, observed at both the cDNA and protein levels as c.207_318del, p.Cys70Glufs*81. The complete deletion of Exon3 leads to a subsequent alteration of the reading frame, producing a truncated 149AA protein. These minigene findings are noteworthy and further support the pathogenic mechanism underlying this disease. We identified novel mutations that not only clarified the etiology of the disease in our patients but also expanded the spectrum of GM3SD mutations. This discovery may offer insights into disease management and genetic counseling.

## Conclusions

We present a case of a Chinese boy with compound heterozygous variants in the *ST3GAL5* gene. This represents the inaugural report of the c.207-1G > T variant, thereby broadening the genetic mutation spectrum of GM3 synthase deficiency. These findings offer valuable insights for genetic counseling and prenatal diagnosis in affected patients.

## Data Availability

The data for this article are not publicly available because of privacy concerns. Requests to access these datasets should be directed to LHQ (hongqian.liu@163.com) or WJ (hhwj_123@163.com).

## Abbreviations

GM3SD: GM3 synthase deficiency
GSLs: Glycosphingolipids
PCR: Polymerase chain reaction
